# Japan Coma Scale and the Disorientation of the Nervous System

**DOI:** 10.2188/jea.JE20220247

**Published:** 2023-10-05

**Authors:** Soichiro Saeki, Yohei Kurosawa, Koichiro Tomiyama

**Affiliations:** Emergency Medicine and Critical Care, Center Hospital of the National Center for Global Health and Medicine, Tokyo, Japan

**Keywords:** Japan Coma Scale, Glasgow Coma Scale, central nervous system, emergency medicine

We read with considerable interest the study by Nakajima et al^1^ to create a conversion of the Japan Coma Scale (JCS) to the Glasgow Coma Scale (GCS). Their findings would surely contribute to promoting studies in the field of emergency medicine in Japan to be reported globally.

As the authors have mentioned, JCS is the most common method to gauge the level of consciousness in Japan.^1^ For example, the paramedics that contact the patient at the site of first response would evaluate the level of consciousness using JCS, instead of GCS. As a result, many databases in Japan extensively record JCS, which is not an evaluation method of use internationally. This has been a limitation of Japanese studies to be compared with other studies from abroad, and the conversion method developed by Nakajima et al^1^ would serve as a crucial link to bring the Japanese evidence to a global level. Furthermore, as the level of consciousness has been noted to alter communication between medical staff and patients, this study may influence studies that are not limited to emergency medicine and epidemiology, such as public health studies focusing on foreign patients requiring linguistic assistance.^2^

However, we also believe that this method requires caution when being utilized, especially in clinical settings. In particular, we would like to emphasize the importance of JCS 1. We have noticed that the conversion table by Nakajima et al^1^ includes both JCS 0 and JCS 1 in GCS 15. JCS 1 is a state where the patient’s consciousness is almost full, awake without any stimuli.^3^ As physicians in an emergency department of a Japanese medical facility, we would like to highlight several points that may be overlooked when this conversion method is used and JCS 0 and 1 are grouped together.

First, under-triage of patients with disorientation of the nervous system may occur. JCS 1 has been noticed to include diagnosis that are known to cause disorientation of consciousness similar to patients assessed to be >1 in JCS.^4^ Thus, this method may overlook the benefits of the JCS to screen very subtle alterations in consciousness.

Second, patients that undergo a minor change in consciousness may be dismissed. There are several diseases that receive treatment in the emergency department that may experience a slight alteration in consciousness. For example, patients with sepsis are known to present a mild disorientation of consciousness, prior to deteriorating to a state of septic shock.^5^^,^^6^ Such changes in consciousness are crucial warning indications in clinical settings, and studies that try to identify such characteristics should use caution when using this conversion method.

Overall, we acknowledge the significance of the study by Nakajima et al^1^ to convert JCS into GCS, especially in database research and epidemiological studies. However, we also believe that JCS is an important evaluation method of awareness in patients, and the JCS and GCS results should also be provided simultaneously. As the authors mention in the limitations, JCS and GCS should be evaluated independently,^1^ and data obtained directly from the evaluation by physicians in contact with the patients should be prioritized over this conversion table, particularly when utilizing data obtained from clinical settings. Researchers conducting database research utilizing the methods by Nakajima et al^1^ should be aware of such limitations.
